# 3D-Printed Continuous Flax Fiber-Reinforced Composites Based on a Dual-Resin System

**DOI:** 10.3390/polym17182515

**Published:** 2025-09-17

**Authors:** Yu Long, Zhongsen Zhang, Zhixiong Bi, Kunkun Fu, Yan Li

**Affiliations:** 1 School of Aerospace Engineering and Applied Mechanics, Tongji University, Shanghai 200092, China; 2 Yongjiang Laboratory, Ningbo 315202, China; 3 Shanghai Institute of Aircraft Mechanics and Control, Shanghai 200092, China

**Keywords:** 3D printing, continuous plant fiber, dual resin system, in situ polymerization, mechanical properties

## Abstract

Compared with traditional continuous plant fiber-reinforced thermoplastic composites, their 3D-printed counterparts offer distinct advantages in the rapid fabrication of complex geometries with integrated forming capabilities. However, the impregnation process of continuous plant fiber yarn with thermoplastic resin presents greater technical challenges compared to conventional synthetic fibers (e.g., carbon or glass fibers) typically employed in continuous fiber composites, owing to the yarn’s unique twisted structure. In addition, low molding pressure during 3D printing makes resin impregnation more difficult. To address the impregnation difficulty within plant fiber yarn during 3D printing, we employed two low-viscosity resins, liquid thermoplastic resin (specifically, reactive methyl methacrylate) and thermosetting epoxy resin, to pre-impregnate flax yarns, respectively. A dual-resin prepreg filament is developed for 3D printing of flax fiber-reinforced composites, involving re-coating pre-impregnated flax yarns with polylactic acid. The experimental results indicate that liquid thermoplastic resin-impregnated composites exhibit enhanced mechanical properties, surpassing the epoxy system by 39% in tensile strength and 29% in modulus, attributed to improved impregnation and better interfacial compatibility. This preparation method demonstrates the feasibility of utilizing liquid thermoplastic resin in 3D-printed continuous plant fiber composites, offering a novel approach for producing highly impregnated continuous fiber filaments.

## 1. Introduction

Continuous fiber-reinforced composites (CFRCs) benefit from the use of high-performance continuous fibers, a compatible resin matrix, and optimized structural design, factors which enable them to perform exceptional specific strength and stiffness [1,2,3]. The resin matrix used can be thermosetting (TS) or thermoplastic (TP). TS resins form a three-dimensional cross-linked network upon polymerization, making them hard to reshape, weld, or recycle easily, unlike TP resins [4]. Consequently, as environmental issues escalate, continuous fiber-reinforced thermoplastic composites, especially those reinforced with plant fibers derived from bio-based materials, are attracting increasing attention and research [5,6,7].

However, the high melt viscosity of TP resins limits their application in continuous fiber-reinforced composites [8], as higher temperatures and pressures are necessary for optimal fiber impregnation. Thus, TP-based CFRCs are mainly prepared by hot compaction, powder impregnation, injection molding, and automated fiber placement to improve impregnation [9,10,11,12]. For complex curved surfaces or porous structures, customized, expensive molds are required. The advent of 3D printing technology streamlines the fabrication of TP-based CFRCs by eliminating the need for molds and post-assembly, thereby significantly reducing manufacturing costs and time [13]. The currently commercialized suppliers of 3D-printed TP-based CFRCs are mainly from Markforged and Anisoprint [14,15,16,17]. The continuous carbon fiber or glass fiber filaments produced by Markforged utilize a thermoplastic resin known as polyamide 6I. This polyamide, characterized by high melt viscosity and hygroscopicity, results in a significant number of voids within the pre-impregnated filament [18]. In contrast, Anisoprint employs epoxy resin, which has a low viscosity that reduces the voids within the pre-impregnated filament. Additionally, the surface of the pre-impregnated filament is coated with thermoplastic resin, serving as an adhesive during printing [17]. This dual resin system, which combines the advantages of both resins, represents a promising method for preparing prepreg filaments, albeit with the need for further consideration of the interfacial compatibility between the two resins. Thermoplastic resin polylactic acid (PLA) is commonly utilized as the resin matrix for continuous plant fiber-reinforced bio-composites in 3D printing, given the limited temperature resistance of plant fibers (~200 °C) [19,20]. However, the high melt viscosity of PLA poses challenges for the impregnation of twisted plant fiber yarns at processing temperatures. Consequently, there is a need to identify a low-viscosity thermoplastic resin.

Elium resin, a reactive methyl methacrylate-based resin developed by Arkema, satisfies the low viscosity requirement. It has low viscosity (100–500 mPa·s), short processing time, and excellent impact resistance [21,22]. It can be transformed into a thermoplastic resin during liquid molding and has been successfully applied in wind turbine blades and boats [23,24]. Furthermore, CFRCs prepared using Elium can be ground or thermally degraded to obtain short fiber composites, fibers, and monomers for reuse. Given its excellent mechanical properties and environmental friendliness, many researchers have investigated its application in continuous fibers [4,25,26,27,28,29], Bhudolia et al. [25,29,30,31] conducted an in-depth study on various properties of carbon fiber/Elium composites, including vibration damping, dynamic mechanical properties, process optimization, flexural strength, and impact resistance. However, there are currently limited studies on the properties of Elium itself, primarily focusing on aging behavior and polymerization kinetics [32]. The low viscosity of Elium facilitates the impregnation of the resin into the yarn. However, due to its fast reaction time and volatile monomers, the use of Elium in open systems for the preparation of impregnated filaments with plant flax yarns poses a new challenge in 3D-printed CFRCs.

In this work, inspired by the design of the dual-resin system, we propose a novel method to prepare impregnated filaments using Elium and PLA for 3D-printed CFRCs. To optimize the resin processing, we conducted rheological, thermal, and thermomechanical analyses of Elium to determine the optimal reaction temperature and time. In an open system, we pre-impregnated flax yarns with liquid Elium and partially polymerized the resin surface using UV LED to prevent dripping during the subsequent heating polymerization process. Subsequently, PLA is coated on the surface of the pre-impregnated flax filaments with thermoplastic or thermoset resins. CFRCs’ samples are then printed. Finally, microscopic analyses and mechanical property tests are carried out to assess the feasibility of using Elium to prepare thermoplastic pre-impregnated filament with flax fiber yarns. Simultaneously, a comparative study is carried out using a low-viscosity epoxy resin.

## 2. Materials and Methods

### 2.1. Materials

Elium and epoxy resins were used for pre-impregnation of flax yarns. Elium^®^ C195 and peroxide blends were recommended and provided by Arkema (Changshu, China). Elium^®^ C195 has a low viscosity (100 mPa·s at 25 °C) and adjustable processing time (>2 h), compared with other types of Elium [21]. Elium^®^ C195 was polymerized to produce high molecular weight methyl methacrylate polymers using peroxide blends as thermal initiators and Omnirad-184 and Omnirad-819 (IGM RESINS) as photoinitiators [33]. The epoxy resin (Epoxy, TECHSTORM^TM^ 481/486S) was provided by TECHSTORM (Shanghai, China), with a mass ratio of 100: 26 for resin to hardener. The densities of Elium^®^ C195 and TECHSTORMTM 481/486S are both 1.15 g/cm^3^.

The flax yarns (Dew retting, Normandy, France) with a linear density of 67 Tex were provided by Zhejiang Jinyuan Flax Co., Ltd., (Jiaxing, China). The flax fiber was twisted with 365 Turn/m, and had a density of 1.5 g/cm^3^. The physical properties of the flax yarns used have been mentioned in previous work [34]. PLA 4032D was purchased from NatureWorks LLC, (Plymouth, MA, USA). The melting temperature and the processing temperature range were 169 °Cand 185–210 °C, respectively. Table 1 shows the physical properties of these resins provided by the manufacturers. Absolute ethanol and silane (triethoxyvinyl silane, A151) were purchased from Sinopharm Chemical Reagent Co., Ltd. (Shanghai, China). Both flax yarn and PLA were thoroughly dried at 80 °C and 90 °C, respectively, before use, to minimize the impact of moisture on the final mechanical properties.

### 2.2. Pre-Impregnation of Flax Yarns

**Figure 1:**
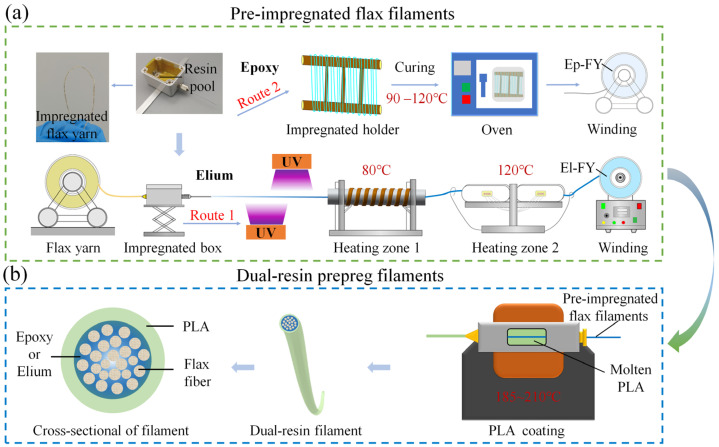
Schematic diagram of the fabrication process for (**a**) pre-impregnated flax yarn filaments and (**b**) dual-resin prepreg filaments. The Elium-impregnated flax yarn was abbreviated as El-FY, while the Epoxy-impregnated flax yarn was abbreviated as Ep-FY.

Pre-impregnation of flax yarns with Elium and Epoxy resin was performed using a customized preparation system, as depicted in Figure 1a. The temperatures of heating zone 1 (HZ1) and heating zone 2 (HZ2) were controlled by the temperature controllers (TC), which were abbreviated as HZ1-TC and HZ2-TC, respectively. The flax yarns were pretreated with A151 silane before use [38]. The impregnated module comprised an inlet nozzle, a resin pool, and an outlet needle. To minimize the friction between the yarns and the inlet nozzle, a Teflon tube was embedded in the inlet nozzle. A longer needle was employed to improve impregnation efficiency by extending the contact time and increasing the pressure between the resin and yarns. The subsequent process was bifurcated into two pathways based on the distinct reactive times of the two resins. The relevant process parameters were listed in Table 2.

In Route 1, two 12 W/cm^2^ UV LEDs (395 nm, H7020, Howsuper (Shenzhen, China)) were positioned facing each other with a 3 cm misalignment. They were placed 1.5 cm away from the flax filament to photocure the Elium on its surface. The UV LEDs have an irradiated area of 70 × 20 mm. This process photocured the outer resin layer, while the resin inside the yarn remained in a liquid state [39]. Since the polymerization reaction of Elium resembled that of polymethyl methacrylate (PMMA), a two-step heating process could prevent explosive polymerization [40]. The preliminary reaction was carried out in the aluminum alloy tube equipped with a heater band, with HZ1 maintained at a temperature of 80 ± 5 °C. Subsequently, the Elium-impregnated flax yarn (El-FY) was transported to the ceramic heating plate HZ2 for high-temperature reaction, with HZ2 maintained at a temperature of 120 ± 5 °C. Following thermoforming, the Elium covering the flax yarns transitioned from a liquid to a solid state. Subsequently, the El-FY was wound up using the winding system. At this pulling speed, the surface of the pre-impregnated yarn remains non-tacky and does not adhere to adjacent layers during winding.

In Route 2, the curing reaction of Epoxy typically requires a longer time (>2 h), necessitating the use of a Teflon cloth-coated wooden holder to secure yarns and prevent adjacent yarn bonding during curing. Initially, excess resin on the Epoxy-impregnated flax yarns (Ep-FY) was removed using a silicone sheet. Subsequently, it was wrapped around the holder and placed in an oven for curing at 90 °C for 1 h and 120 °C for 2 h. Finally, the cured and cooled Ep-FY were wound up and prepared for further use.

### 2.3. Preparation of Dual-Resin Prepreg Filaments

The dual-resin prepreg filament was prepared by re-coating pre-impregnated flax filament with PLA resin, as shown in Figure 1b, using a method similar to that described for pre-impregnated filaments in previous work [34]. Cured Epoxy could not be remelted and could only be softened, so a larger nozzle (0.8 mm) was selected to prevent damage to the flax yarn. The cross-section of the filament was composed of an inner layer of Epoxy or Elium-impregnated yarns and an outer layer of PLA resin. Furthermore, the use of pre-impregnated flax filaments eliminated the need for further impregnation of the yarn during 3D printing, allowing for an increase in the PLA coating pulling speed from 1.2 m/min to 2.4 m/min, thereby enhancing productivity.

### 2.4. Three-Dimensional Printing of CFRCs

The dual-resin prepreg filaments were fabricated into CFRCs using a customized co-extrusion 3D printing system [34]. The printing parameters included a line width of 0.4 mm and a layer height of 0.25 mm. The printing temperature was set to 205 °C. The printing speed was set at 7.5 mm/s, and the printing path for the composite structure was designed using open-source Cura V4.1 software. All samples were printed using a rectilinear filling pattern oriented at 0° (longitudinal). The fiber volume fraction (*v_f_*) of PLA-coated El-FY (PLA-El-FY) and PLA-coated Ep-FY (PLA-Ep-FY) were 38.2% and 32.4%, respectively.

### 2.5. Rheological Property

The rheological properties of the three resins—Elium, Epoxy, and PLA—were evaluated using a Haake Mars rheometer (Thermo Scientific, Waltham, MA, USA) equipped with 20 mm parallel plates and a 1 mm gap. Viscosity measurements were conducted using frequency sweeps at various temperatures, as detailed below: (1) Shear rate ranged from 1 s^−1^ to 20 s^−1^ at 25 °C. (2) The temperature was set to 205 °C, with a shear rate of 0.5 s^−1^. (3) The complex viscosity of the resin was tested using the oscillation mode within a temperature range of 25–240 °C, at a frequency of 1 Hz and a heating rate of 5 °C/min.

### 2.6. Differential Scanning Calorimetry (DSC)

The thermal properties of Elium and Epoxy were investigated using a DSC Q20 instrument from TA Instruments (Newcastle, DE, USA). Approximately 5 mg of each sample was weighed and placed in an aluminum pan. The reaction time at constant temperature test was as follows: (1) Heating scans were recorded at a rate of 100 °C/min until reaching 60 °C, 90 °C, and 120 °C. (2) The samples were maintained at 60 °C, 90 °C, and 120 °C for 30 min. The test for resin polymerization or curing involved recording heating scans at a rate of 10 °C/min from 25 °C to 200 °C. The typical procedure for determining the T_g_ involved: (1) Recording heating scans at a rate of 20 °C/min from 25 °C to 200 °C. (2) The sample was maintained at 200 °C for 3 min to eliminate thermal history, followed by cooling to −10 °C at a rate of 10 °C/min and holding for 3 min. (3) The sample was reheated at a rate of 10 °C/min to 200 °C.

### 2.7. Dynamic Mechanical Analysis (DMA)

A dynamic mechanical analyzer DMA Q800, TA Instruments, (New Castle, DE, USA) was employed to measure the dynamic mechanical behavior of resins and prepreg filaments. The temperature range was set between 25 °C and either 160 °C or 200 °C, with a test frequency of 1 Hz and a heating rate of 3 °C/min. The dimension of the resin samples was 30 × 5 × 1.5 mm (length × width × thickness). The prepreg filaments had a length of 40 mm and a diameter ranging from 0.33 mm to 0.35 mm.

### 2.8. Micro-Morphology Characterization

Surface morphologies of the flax yarns, fracture surfaces of filaments, and composites were detected by SEM (EVO18 from Carl Zeiss, Oberkochen, Germany) at an accelerating voltage of 20 kV. To improve conductivity, a thin layer of platinum was deposited on the surface of each sample. SEM samples of pre-impregnated flax and dual-resin prepreg filaments were obtained through liquid nitrogen quenching.

### 2.9. Fiber Volume Fraction and Void Content

A density balance was used to measure the weight of the sample in air (*m_air_*) and the weight in ethanol (*m_ethanol_*) to determine the volume of the 3D-printed composites (*v_3DPc_*). The fiber volume fraction of 3D-printed composites (*v_f_*) was calculated using the linear density (*tex*), the length of flax yarns used (*l_f_*), and the density of flax yarn (*ρ_f_*). The *v_f_* was calculated according to the following formulas:

(1) $$v_{3\mathrm{DPc}}=\;\frac{m_{air}-m_{ethanol}}{\rho_{ethanol}}$$

(2) $$v_{f}=\frac{l_{f}\times tex\times {\rho_{f}}^{-1}}{v_{3DPc}}$$

The void content has been described in previous work [38].

### 2.10. Mechanical Properties

The prepreg filaments were tested using a 5 kN load cell (Tinius Olsen 100ST, Philadelphia, PA, USA) at a loading rate of 2 mm/min. The gauge length for the prepreg filament was 20 cm according to ISO 10618:2004 [41]. The tensile modulus was acquired with a strain range of 0.05% to 0.1%, as suggested by Shah [42], to consider the non-linear tensile behavior of flax fibers at the (sub)microscale. Tensile tests were conducted on CFRCs in accordance with the ASTM D3039 standard, using a universal testing machine (Tinius Olsen 100ST, USA) at a loading rate of 2 mm/min. The samples were equipped with an extensometer featuring a 50 mm gauge length to monitor elongation during testing. The dimension of 3D-printed CFRCs was 150 × 8 × 1 mm^3^. Three-point bending tests were also performed using a universal mechanical testing machine (Tinius Olsen 100ST, USA) at a crosshead speed of 2 mm/min. The span-to-thickness ratio was set as 16:1 according to the ASTM D7264 standard [43]. The dimension of 3D-printed CFRCs was 100 × 13 × 2 mm^3^. A minimum of five samples were tested for the CFRC group, and ten samples were tested for the prepreg filament group.

## 3. Results and Discussion

### 3.1. Rheological Properties of Elium and Epoxy Resins

**Figure 2:**
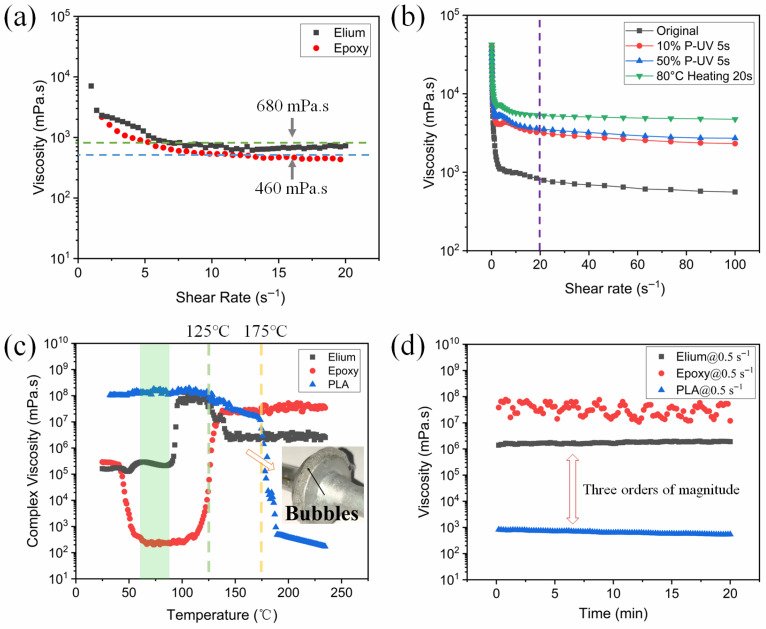
The viscosity of (**a**) Elium and epoxy at shear rates of 0–20 s^−1^ at 25 °C and (**b**) Elium subjected to different curing treatments, “10% P-UV” and “50% P-UV” denote 10% and 50% UV power exposure, respectively. The complex viscosity of (**c**) Elium, epoxy, and PLA from 25 °C to 240 °C, the inset photo depicts the bubbles formed in Elium after testing. The green zone indicates the viscosity difference between Elium and epoxy before rapid reaction. (**d**) Elium, epoxy, and PLA at 205 °C with a shear rate of 0.5 s^−1^.

The variation in viscosity of Elium and Epoxy for shear rate is shown in Figure 2a. Despite the manufacturer’s data indicating that Elium resin has half the viscosity of epoxy resin (Table 1), rheometer measurements reveal comparable viscosities for these two resins at 25 °C. Figure 2b illustrates the variation in viscosity of Elium under different treatments. Notably, UV irradiation effectively increases the initial viscosity of Elium from 790 mPa·s to 3532 mPa·s at 20 s^−1^, thereby preventing resin dripping during the impregnation process. The viscosity of Elium does not significantly change with varying UV irradiation intensities, as demonstrated in Figure S2. After heat treatment with HZ1 (80 °C for 20 s), the viscosity of Elium can be further increased, yet it remains in a liquid state.

Figure 2c depicts the resin viscosity across a specific temperature range. From 40 °C to 150 °C, the complex viscosity of Elium exhibits an initial increase followed by a decrease with temperature, whereas epoxy displays the opposite trend. In their uncured state, Elium and epoxy demonstrate significantly reduced viscosity relative to molten PLA, providing a distinct rheological advantage for achieving thorough intra-yarn fiber bundle impregnation in flax fiber composites. Within the temperature range of 65 °C to 80 °C (green zone), the viscosity of the two liquid resins differs significantly by approximately three orders of magnitude before rapid reaction. The complex viscosity of Elium and epoxy can be divided into three stages. For Elium, the first stage spans from 25 °C to 88 °C, during which the complex viscosity experiences minor fluctuations. This phenomenon may be attributed to the partial volatilization of monomers within Elium as the temperature rises. The second stage, from 88 °C to 145 °C, witnesses a dramatic change in complex viscosity. A stable polymerization state is observed within the temperature range of 97 °C to 126 °C, forming a plateau region, with the maximum complex viscosity of Elium reaching approximately 108 mPa·s. Subsequently, the complex viscosity gradually decreases from 126 °C to 147 °C, attributed to the gradual disappearance of the generated bubbles, which reduces the internal pressure of the resin. The inset photo in Figure 2c reveals a significant number of bubbles surrounding the molds of Elium. The final stage spans from 147 °C to 240 °C, during which the complex viscosity of Elium remains stable at approximately 3.0 × 10^6^ mPa·s. For epoxy, its complex viscosity remains stable during the first stage (25–40 °C). In the second stage, spanning from 40 °C to 139 °C, the complex viscosity of the epoxy undergoes a process of sharp decrease, followed by stabilization and then a rapid increase. Particularly within the temperature range of 40 °C to 66 °C, the complex viscosity of epoxy decreases from 2.4 × 10^5^ mPa·s to 239 mPa·s. A stable viscosity range of 200–300 mPa·s is observed between 66 °C and 103 °C, which is highly suitable for the impregnation of fibers or fabrics. Above 103 °C, the complex viscosity increases rapidly, signifying the accelerated progression of the curing reaction. The third stage spans from 139 °C to 245 °C, during which the curing reaction nears completion, resulting in a stable complex viscosity for the epoxy. The fully cured epoxy resin exhibits a higher complex viscosity compared to Elium. Additionally, for PLA, its molecular chain movement becomes evident at 125 °C, initiating a gradual decline in viscosity. Upon exceeding the melting point at around 165 °C, the complex viscosity of PLA decreases rapidly [44]. Despite the complex viscosity of only 400 mPa·s at 200 °C, PLA faces challenges related to the heat resistance of plant fibers and its thermal stability at higher molding temperatures.

To determine the viscosity of the three resins within the printing head, a shear rate of 0.5 s^−1^ (corresponding to a linear speed of 5 mm/s) is set at 205 °C to observe the melt viscosity of Elium, epoxy, and PLA, as depicted in Figure 2d. After polymerization, Elium exhibits a relatively high melt viscosity, which is three orders of magnitude greater than that of PLA. This implies that PLA, serving as the outer layer, will not penetrate the Elium impregnated yarn during printing. The softened epoxy displays significant viscosity fluctuations, which are attributed to the uneven and rigid surface of the test sample. The viscosity of softened epoxy is greater than that of both Elium and PLA resins.

### 3.2. Thermal Properties of Elium and Epoxy

**Figure 3:**
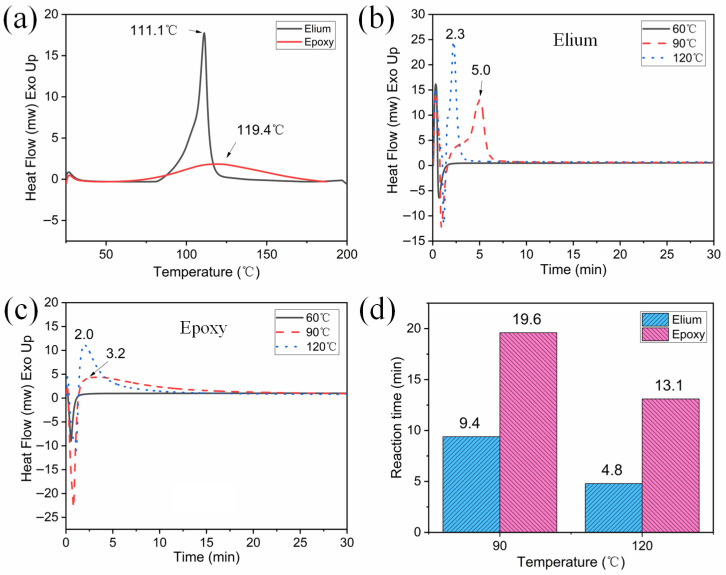
The thermal properties of (**a**) the reaction temperature range of Elium and epoxy from 25 to 200 °C. The reaction times of (**b**) Elium and (**c**) epoxy at different temperatures, along with the comparison of (**d**) their overall reaction times at 90 °C and 120 °C.

In order to determine the optimal polymerization or curing temperatures for preparing pre-impregnated flax filaments with Elium or epoxy, DSC tests within the range of 25–200 °C are conducted, as illustrated in Figure 3a. The curing temperature range for epoxy spans from 55 °C to 180 °C, exhibiting a gentle exothermic curve with a peak at 119.4 °C. Compared with epoxy, Elium undergoes a more vigorous polymerization reaction within a narrower temperature range of 76 °C to 125 °C, with an additional 13% of exothermic enthalpy value (Table S1). The exothermic peak of Elium occurs at 111.1 °C, characterized by its high and sharp intensity.

Temperature and time are both crucial factors influencing the reaction degree of resins. The reaction times for Elium and epoxy at various temperatures are depicted in Figure 3b,c, respectively. Neither Elium nor epoxy can undergo polymerization or curing at 60 °C within 30 min. At 90 °C and 120 °C, the two resins exhibit distinct exothermic peak shapes. Elium shows a high and narrow peak, while epoxy displays a low and broad peak with a pronounced tailing. At both temperatures, although the exothermic peak of epoxy appears slightly earlier than that of Elium, the overall exothermic duration is longer for epoxy, indicating that it requires more time to complete curing. The overall reaction times of the two resins at 90 °C and 120 °C are shown in Figure 3d. At 90 °C, Elium undergoes polymerization within 9.4 min, whereas epoxy requires approximately 20 min for curing. At 120 °C, the polymerization time of Elium is only half that of 90 °C, whereas epoxy’s curing time at this temperature is shortened to 13 min. Based on the above rheological and thermal analysis, combined with the reaction temperature recommended by the suppliers in Table 1, 80 °C and 120 °C are recommended as the optimal heating temperatures for ZH1 and ZH2, respectively. Excessively high initial heating temperature (ZH1) may cause a rapid viscosity increase and the explosive polymerization of the Elium resin, negatively impacting the impregnation of flax yarn.

### 3.3. Thermo-Mechanical and Tensile Properties of Pre-Impregnated Flax Filaments

**Figure 4:**
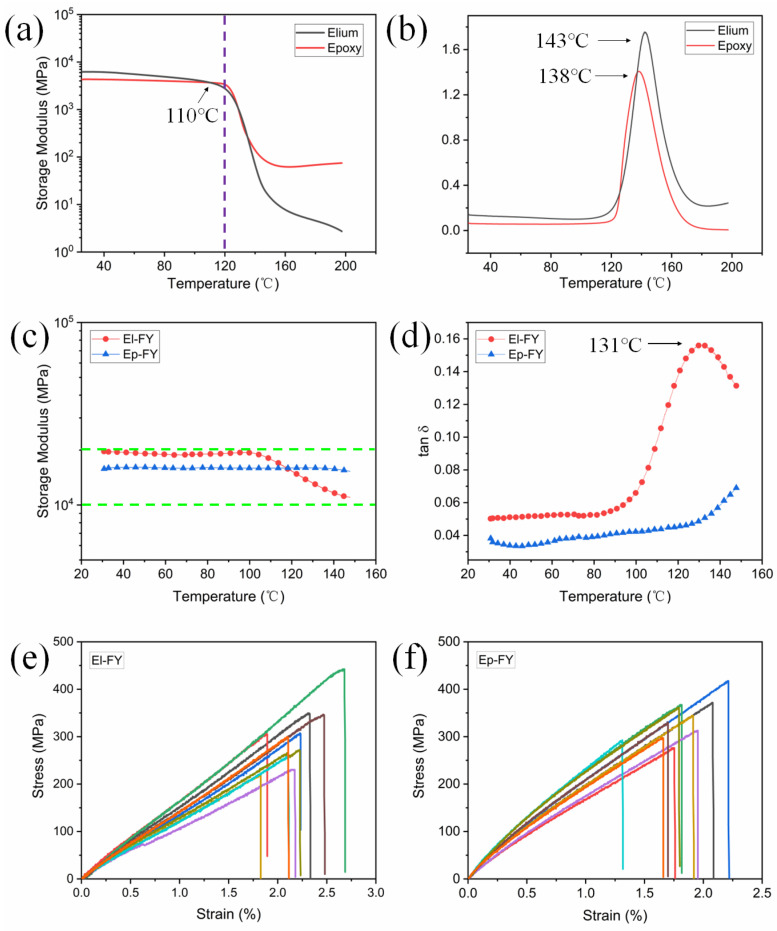
The dynamic mechanical analysis curves of resins and the pre-impregnated flax filaments: (**a**,**c**) storage modulus, (**b**,**d**) tanδ. The green dotted lines indicate the range of the storage modulus for the two samples. The stress–strain curves of (**e**) El-FY and (**f**) Ep-FY. The different color lines represent different test samples.

The initial storage modulus of Elium is higher than that of epoxy by 200 MPa, but the storage modulus of Elium gradually decreases, as shown in Figure 4a. After 110 °C, the storage modulus of epoxy exceeds that of Elium, which is attributed to the stable crosslinked network structure of epoxy. The T_g_ of Elium is slightly higher than that of epoxy, as shown in Figure 4b. The thermo-mechanical performance curves for El-FY and Ep-FY are depicted in Figure 4c. The storage moduli of El-FY and Ep-FY are higher than that of the pure resin due to the reinforcing effect and the suppression of resin flow by the fibers. Within the temperature range of 30 °C to 100 °C, the storage modulus of El-FY exhibits superior stability compared to that of pure Elium. A significant decline in the storage modulus of El-FY is observed after surpassing 110 °C. This may limit its applications in high-temperature or load-cycling scenarios within rail transportation or automotive sectors. Specifically, the storage modulus of El-FY decreases by 43% when the temperature reaches 150 °C. In contrast, the storage modulus of Ep-FY remains stable throughout the entire temperature range from 30 °C to 150 °C. This difference can be attributed to the fact that once the T_g_ is reached, the molecular segments of the thermoplastic resin begin to move more freely, leading to a rapid decrease in storage modulus. Although the T_g_ of the epoxy resin is also attained, its molecular segment mobility is restricted by the crosslinked network structure, resulting in less pronounced movement. This phenomenon is further corroborated by the tanδ curves presented in Figure 4d, where the slope of the El-FY curve undergoes a sharp change at 90 °C, while the slope of the Ep-FY curve changes at 130 °C. The T_g_ of Elium in El-FY is determined to be 131 °C via DMA.

To further investigate the mechanical properties of the two resin-impregnated yarns, the stress–strain curves of the El-FY and Ep-FY are shown in Figure 4e,f. Although the maximum tensile stress of El-FY is slightly higher than that of Ep-FY, both filaments exhibit tensile stresses primarily around 300 MPa. Additionally, El-FY demonstrates better elongation at break and more uniform dispersion compared to Ep-FY, highlighting the superior toughness of Elium.

### 3.4. Micro-Morphology Analysis for Filaments

#### 3.4.1. Pre-Impregnated Flax Filaments

**Figure 5:**
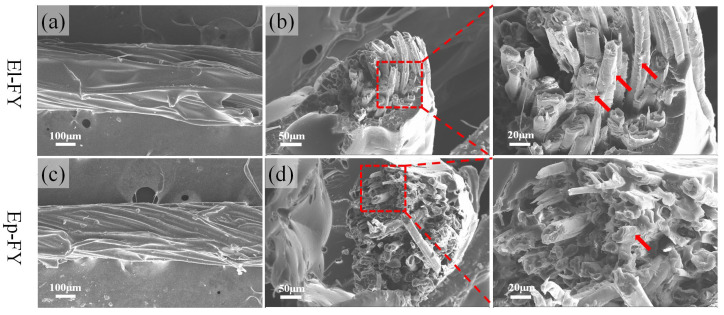
The surface morphologies of (**a**) El-FY and (**c**) Ep-FY and the fracture morphologies of (**b**) El-FY and (**d**) Ep-FY subjected to cryogenic embrittlement. The red arrow indicates the resin residue on the fiber surface.

Figure 5a,c display the micrographs of El-FY and Ep-FY. The contours of the fibers in the outer layer of the yarn are distinctly visible, suggesting that the outer layer of resin in the pre-impregnated flax filament is quite thin. The diameters of El-FY and Ep-FY are 348 ± 85 μm and 332 ± 41 μm, respectively. The two types of pre-impregnated flax filaments show distinct fracture cross-sections, as shown in Figure 5b,d. The fracture cross-section of El-FY appears uneven, with most fibers being pulled out of the matrix. This suggests that the Elium resin did not fully penetrate the center of the yarn. However, in the enlarged image, residual resin is observed on the fibers, indicating good interfacial bonding between the Elium resin and the fibers. In contrast, the cross-section of the epoxy-impregnated yarn is relatively flat, showing a more obvious brittle fracture. The enlarged fracture image reveals that the resin has penetrated the interior of the yarn, with fiber breakage being the predominant mode of failure.

#### 3.4.2. Dual-Resin Prepreg Filaments

**Figure 6:**
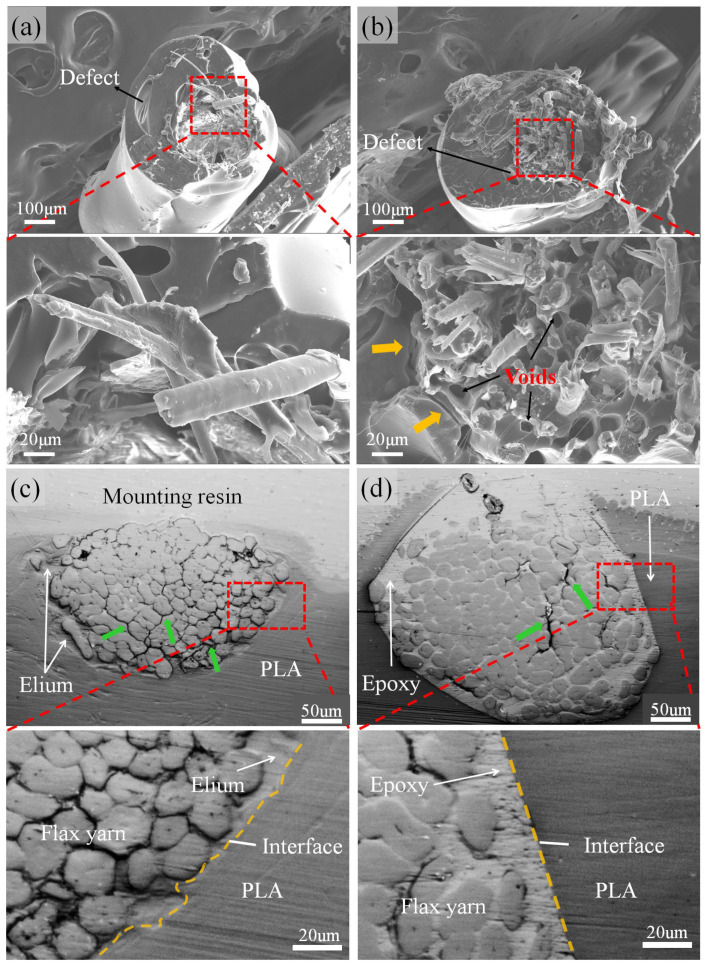
The fracture morphologies of (**a**) PLA-El-FY and (**b**) PLA-Ep-FY subjected to cryogenic embrittlement. The orange arrows indicate the interface cracks between PLA and pre-impregnated flax filament. The polished sections of (**c**) PLA-El-FY and (**d**) PLA-Ep-FY. The green arrows indicate the cracks in flax filaments.

Ep-FY, which contains cured epoxy, cannot be directly printed. The content of Elium resin in El-FY is also insufficient for printing. Therefore, both pre-impregnated flax filaments are coated with PLA resin for 3D printing. The cross-sectional SEM images of PLA-El-FY and PLA-Ep-FY are shown in Figure 6a,b. Due to the increased pulling speed of the PLA coating, some void defects appear in the outer layer of both PLA-El-FY and PLA-Ep-FY. As depicted in the enlarged image, although the majority of the yarns in the PLA-El-FY composite were pulled out, the interfacial bonding between the outer flax fiber and the PLA layer remains strong. This is attributed to the good compatibility between Elium and PLA [45]. In the enlarged fracture morphology image of PLA-Ep-FY, many voids and broken fibers are observed inside the filaments. Some of these voids originate from the pull-out fibers, while others are air bubbles introduced during epoxy impregnation. Additionally, these voids may also originate from residual water vapor within the yarn, which stems from the hygroscopic nature of flax yarn. The presence of voids weakens the mechanical properties of CFRCs. An obvious crack (indicated by orange arrows) between the pre-impregnated flax filament and the PLA-coated layer is observed, indicating weak interfacial bonding between epoxy and PLA. This weak interface affects stress transfer between the pre-impregnated flax filament and the PLA resin. Comparing the enlarged images of PLA-El-FY and PLA-Ep-FY, it is evident that low-viscosity epoxy resin penetrates more easily into the center of the flax yarn at 50–80 °C. The polished cross-sections of PLA-El-FY and PLA-Ep-FY, as shown in Figure 6c,d, cracks are primarily observed in the outer layer of yarns in PLA-El-FY and the inner part of yarns in PLA-Ep-FY, corresponding to the fracture morphologies of the samples. From the enlarged images in Figure 6c,d, it can be observed that compared to the interface between the two resins in PLA-El-FY, the interfacial boundary in PLA-Ep-FY is more distinct. This indicates that the interfacial compatibility between epoxy resin and PLA is weaker than that between Elium and PLA, making it more prone to cracking under external force. These results further support the discussion on impregnation and interfacial compatibility.

### 3.5. Fiber Volume Fraction and Void Content of 3D-Printed Composites

**Figure 7:**
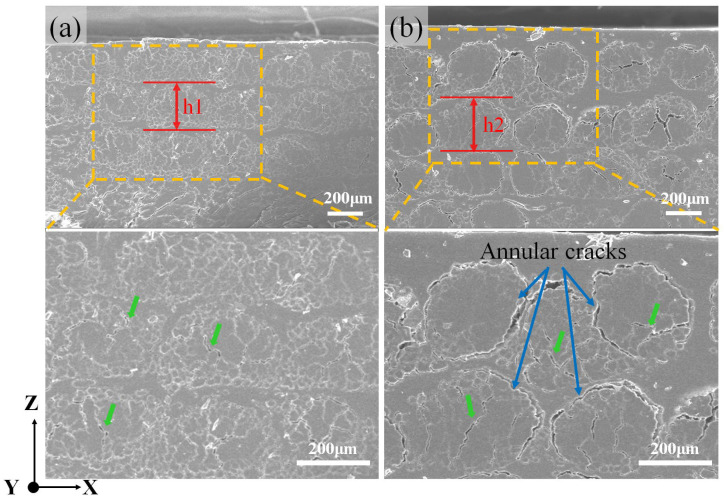
The SEM images of the polished cross-sections of (**a**) PElFCs and (**b**) PEpFCs. h1 and h2 indicate the average height of the yarns in the Z-direction. The enlarged images show the internal cracks in flax yarns (green arrows) and the interfacial annular cracks of PEpFCs (blue arrows).

The composite samples printed using PLA-El-FY and PLA-Ep-FY are named PElFCs and PEpFCs, respectively. The *v_f_* of PElFCs and PEpFCs can be obtained by formulas (1) and (2) [28]. As shown in Table 3, the *v_f_* of PEpFCs is 10% lower than that of PElFCs. This can be attributed to the fact that the cured epoxy cannot be remelted during printing. Consequently, the deformation of Ep-FY is minimal under the pressure exerted by the printing nozzle, resulting in no excess resin and internal voids within the yarns being extruded. As a result, the PEpFCs contain more resins and air bubbles. To verify the advantages of the dual-resin system, 3D-printed PLAFCs were also prepared using PLA-FY filaments under the same process conditions, including the parameters for PLA-coated filaments and printing. Due to the absence of low-viscosity resin impregnation, the high-speed coating of PLA resulted in numerous voids within the yarn, leading to a lower fiber volume fraction and impregnation degree in PLAFCs. A quantitative analysis of the decline in *v_f_* was conducted through SEM images of the polished cross-sections of PElFCs and PEpFCs [38]. The cross-sections of the yarns in PElFCs are elliptical, while those in PEpFCs are more circular (Figure 7a,b). The heights of the cross-sections with 50 yarns were calculated for both PElFCs and PEpFCs, revealing that the average height of yarns in PEpFCs (h2) is significantly larger than that in PElFCs (h1), by approximately 41 μm, as detailed in Table 3. The value of h2 exceeds the designated printing layer height of 0.25 mm, indicating a higher resin content. From the enlarged images, some cracks can be observed within the filaments of both samples, which are likely generated when pre-existing defects in the impregnated yarns are subjected to shear stress during polishing. Additionally, due to the poor interfacial compatibility between epoxy and PLA, some Ep-FY debond from the PLA matrix during polishing (Figure 7b). An enlarged image shows interfacial annular cracks around the cross-sections of the Ep-FY, which will affect stress transfer.

**Figure 8:**
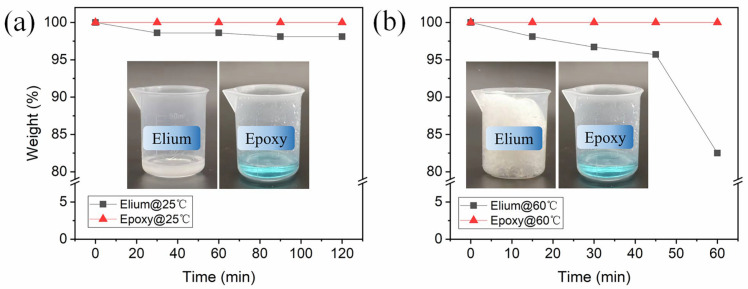
The volatilization test of the resin weight-time relationship: (**a**) at 25 °C and (**b**) at 60 °C, the inset photos show the state of the two resin samples at 25 °C after 2 h and 60 °C after 1 h.

The void contents of PElFCs and PEpFCs are 3.6 ± 0.9% and 5.0 ± 2.0%, respectively. There are no obvious voids in the matrix resin of the polished PElFCs and PEpFCs, indicating that the voids in the PLA portion of the dual-resin pre-impregnated filaments can be effectively eliminated during printing. The impregnation degree of the flax yarn by Elium and epoxy resin was calculated to be 38.2% and 32.8%, respectively, using the weight method [40]. Under the current preparation process, no resin achieved a 50% impregnation degree with the flax yarn. This result is below the expected design value. For Elium, the possible reasons are its volatility in the air during impregnation. The results of the volatilization experiment prove that Elium has high volatility, while epoxy is almost non-volatile (Figure 8, more details refer to Supporting Information). At 25 °C, the weight of the Elium sample decreased by 1.9% after 2 h, with no significant change in the state of the resin (inset photos of Figure 8a). Therefore, designing a long-channel impregnation chamber to extend the impregnation time of Elium resin at room temperature represents an effective method for enhancing the impregnation degree. At 60 °C, the weight of the Elium sample decreased by 17.5% after 1 h. The results show that the monomers in Elium resins have high volatility during HZ1, which affects the impregnation degree of Elium in the pre-impregnated flax filaments. Furthermore, the inset photos of Figure 8b demonstrate that explosive polymerization occurred in Elium due to heat accumulation. Therefore, during processing in an open system, the volatility and rapid polymerization of Elium resin can generate certain amounts of gas, leading to void formation within the impregnated yarn or at the interface between the yarn and the resin, thereby reducing the impregnation quality and interfacial adhesion of the pre-impregnated flax filaments. For epoxy resin, the lower impregnation degree may be attributed to the lower viscosity during the first heating stage (HZ1), where only a small amount of resin remains within the yarns under gravity. Moreover, the introduction of air bubbles during the impregnation process may also be a contributing factor (Figure S3).

### 3.6. Tensile and Flexural Properties of 3D-Printed Composites

**Figure 9:**
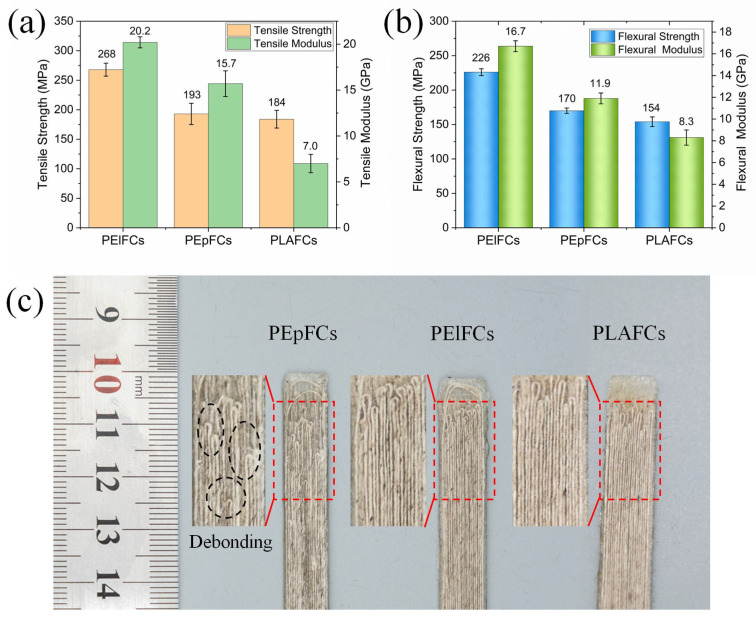
The tensile (**a**) and flexural (**b**) properties of PElFCs, PEpFCs, and PLAFCs. (**c**) Photos of their tensile samples and enlarged end view (inset), with yarn debonding indicated by the black dashed box.

Despite a 10% difference in *v_f_* between PElFCs and PEpFCs, their mechanical properties remain comparable under identical printing conditions. As depicted in Figure 9a,b, PElFCs exhibit superior tensile and flexural properties compared to PEpFCs. Specifically, the tensile strength and modulus of PElFCs are 39% and 29% higher, respectively, while the flexural strength and modulus are 33% and 40% higher, respectively. This can be attributed to the stronger interfacial bonding between the Elium resin and the PLA resin in PElFCs. The poor interfacial compatibility between the two resins is also reflected in the forming quality of the printed samples (Figure 9c). Taking the tensile samples as an example, the ends of PLAFCs and PElFCs samples exhibit a relatively intact shape, while the surface of PEpFCs shows more filaments debonding (black dashed boxes), which makes them more prone to interlayer interface failure. When using a new type of resin to impregnate continuous fibers for preparing printing filaments and 3D-printed composites with a multi-resin system, it is crucial to consider the compatibility between different resins and between resins and fibers. In contrast to PElFCs and PEpFCs, PLAFCs do not exhibit interfacial compatibility issues among different resins. However, the higher void content markedly reduces the mechanical properties of composites. The tensile strength and modulus of PLAFCs are only 69% and 35% of those of PElFCs, respectively, while the flexural strength and modulus are only 68% and 50% of those of PElFCs, respectively. This demonstrates that the dual-resin system strategy effectively enhances the mechanical performance of the 3D-printed composites by increasing the impregnation degree of the flax yarn. Furthermore, due to differences in interfacial compatibility, the actual printed dimensions of PElFCs and PEpFCs may deviate from the designed specifications. Taking the flexural samples as an example, dimensional variations along the length, width, and height directions can reach 6–20% (Figure S2).

## 4. Conclusions

In this work, we employ a novel liquid thermoplastic resin (Elium) to fabricate the pre-impregnated flax yarn filament through a self-designed impregnation, photocuring, and thermo-curing system, exploring the potential of Elium resin for 3D-printed continuous fiber composites. The photocuring treatment effectively controls the viscosity of the resin and prevents dripping. The two-stage temperature increase design effectively prevented excessive polymerization. Dual-resin prepreg filaments are proposed by re-coating the pre-impregnated flax yarns with PLA, which exhibited good printability. When the *v_f_* is 42.3%, the initial impregnation degree of PElFCs is 38.2%, and their tensile strength and modulus reach 268 MPa and 20 GPa, respectively. Compared with pure PLA, PElFCs exhibit a 4-fold increase in tensile strength and a 4.8-fold increase in tensile modulus.

Elium exhibits low viscosity, enabling good impregnation. Regarding thermal properties, Elium offers the advantages of a high reaction rate and high glass transition temperature, which improve productivity and heat resistance in plant fiber-reinforced composites. The tensile properties and stability for pre-impregnated filaments with Elium are better than those with epoxy due to the excellent toughness of Elium itself. Microscopic analysis reveals that Elium effectively impregnates flax yarns. Furthermore, Elium exhibits better compatibility with PLA compared to epoxy, resulting in enhanced interfacial bonding in continuous fiber-reinforced composites. Based on the features of a complete carbon cycle, recyclability, and dual glass transition temperatures, combined with 3D printing technology, this flax yarn-reinforced dual-resin composite holds promise for applications in rail transportation, automotive interiors, sports equipment, and thermally induced deformable robots.

For future work, extended room-temperature impregnation of Elium resin will be explored to further improve the impregnation degree of plant fiber yarns. Meanwhile, the introduction of additional temperature zones to achieve graded temperature control for the Elium resin can effectively reduce the occurrence of explosive polymerization. The reaction heat and volatility of Elium resin in open environments will also be further investigated and controlled to enable the preparation of 3D-printed CFRCs using fully Elium-based resin systems.

## Figures and Tables

**Table 1:** The physical properties of Elium^®^ C195, TECHSTORM^TM^ 481/486S, and PLA 4032D [21,35,36,37].

Properties	Elium^®^ C195	TECHSTORM^TM^ 481/486S	PLA 4032D
Tensile strength (MPa)	66	70–80	53
Tensile modulus (GPa)	3.2	3.0–3.5	3.5
Elongation at break (%)	2.8	4.0–6.0	6.0
Flexural strength (MPa)	111	120–130	/
Flexural modulus (GPa)	2.9	3.0–3.5	/
Glass transition temperature (°C) ^1^	114	140	60
Reaction temperature (°C)	80–115	60–105	/
Reaction time (min)	2–5	>120	/
Viscosity ^2^ (mPa·s at 25 °C)	100	200–300	/

^1^ The values of glass transition temperature (T_g_) were obtained from our lab at second heating curves in a differential scanning calorimetry (DSC) test in Figure S1 and Table S1. ^2^ Viscosity is measured by a coaxial double-cylinder rotational viscometer.

**Table 2:** Processing parameters for the fabrication of pre-impregnated flax yarn filaments.

	Pre-Impregnated Flax Yarn Filaments
Nozzle size (mm)	0.6 (diameter) × 13 (length)
Needle size (mm)	0.72 (diameter) × 50 (length)
Impregnated box length (mm)	52
Heating zone 1 (°C)	80 ± 5
Heating zone 2 (°C)	120 ± 5
Heating zone 1 Length (mm)	600
Heating zone 2 Length (mm)	500
Pulling speed (cm/min)	18

**Table 3:** The *v_f_*, void content, impregnation degree, and the height of flax yarn in PElFCs and PEpFCs.

Samples	*v_f_* (%)	Void Content (%)	Impregnation Degree (%)	Height of Flax Yarn (μm)
PElFCs	42.3 ± 2.6	3.6 ± 0.9	38.2 ± 1.4	222 ± 15
PEpFCs	32.4 ± 1.6	5.0 ± 2.0	32.8 ± 1.6	263 ± 38
PLAFCs	30.9 ± 0.4	12.0 ± 1.0	19.9 ± 2.4	234 ± 24

## Data Availability

The original contributions presented in this study are included in the article and Supplementary Materials. Further inquiries can be directed to the corresponding author.

## Supplementary Materials

The following supporting information can be downloaded at: https://www.mdpi.com/article/10.3390/polym17182515/s1, Figure S1: The DSC curves of Elium^®^ and epoxy resin: (a) second heating, (b) cooling; Figure S2: Photos of the UV-curing resin device: (a) The UV-curing device and sample placement details, and (b) the UV-curing device during operation; Figure S3: (a) The schematic diagram of epoxy impregnated flax yarn in the impregnated box, (b) the photo of epoxy impregnated flax yarn in the impregnated box. Figure S4: (a) Schematic diagram illustrating the dimensional measurement locations for PElFCs, PEpFCs and PLA samples. (b) The photos of PElFCs, PEpFCs and PLA samples. (c) Comparison of sizes among PElFCs, PEpFCs and PLA samples; Table S1: Thermal transition data of Elium^®^ and epoxy resin; Table S2: The deviation in size of PElFCs and PEpFCs compared to pure PLA and design size.

## Abbreviations

The following abbreviations are used in this manuscript:

CFRCs	Continuous fiber-reinforced composites
PLA	Polylactic acid
HZ	Heating zone
TC	Temperature controller
El-FY	Elium-impregnated flax yarn
Ep-FY	Epoxy-impregnated flax yarns
PLA-El-FY	PLA-coated El-FY
PLA-Ep-FY	PLA-coated El-FY
PElFCs	Composite samples printed using PLA-El-FY
PEpFCs	Composite samples printed using PLA-Ep-FY
DSC	Differential Scanning Calorimetry
DMA	Dynamic mechanical analysis
SEM	Scanning electron microscopy
