# Supplementary material for: 3D-Printed Continuous Flax Fiber-Reinforced Composites Based on a Dual-Resin System
Source: Polymers (Basel). 2025 Sep 17;17(18):2515. doi: 10.3390/polym17182515 (PMC12473954; doi:10.3390/polym17182515)
Supplement: Supplementary file 1 [file polymers-17-02515-s001.zip › polymers-3857604-supplementary.pdf]

3D printed continuous flax fiber reinforced composites based on a dual-resin system

Yu Long[1,2], Zhongsen Zhang[1,3,*], Zhixiong Bi[1,3] Kunkun Fu[1,3], Yan Li [1,3,]

[1] School of Aerospace Engineering and Applied Mechanics, Tongji University, Shanghai 200092, China

[2] Yongjiang Laboratory, Ningbo 315202, China

[3] Shanghai Institute of Aircraft Mechanics and Control, Shanghai 200092, China

[*]Correspondence: zhangzhongsen@tongji.edu.cn

1. The thermal performance of Elium and epoxy resin

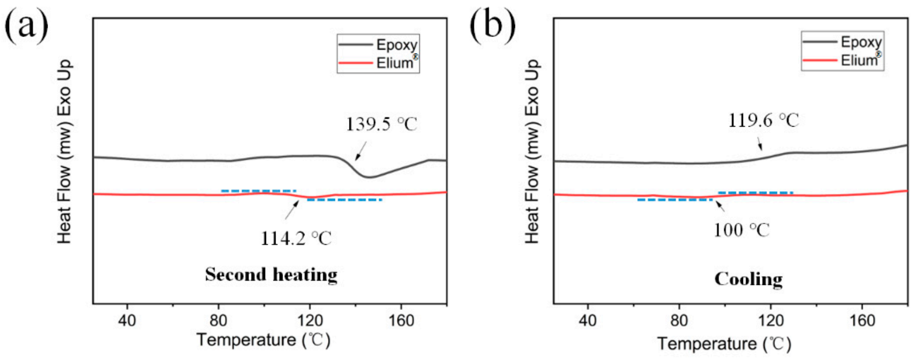

Figure S1. The DSC curves of Elium® and epoxy resin: (a)second heating, (b)cooling.

Table S1. Thermal tansition data of Elium® and epoxy resin[a]

	T_g (°C)	T_{gc} (°C)	ΔH_e (J/g)
Elium®	114.2	100	171.9
epoxy	139.5	119.6	150.1

[a]Scanning rate: 10°C/min, T_g is abbreviations for glass transition temperature at second heating, T_{gc} is abbreviations for glass transition temperature at cooling, ΔH_e is abbreviations for exothermic enthalpy.

2. UV Curing experiment

The curing test device for Elium resin containing photoinitiators under UV irradiation of different intensities was shown below. After curing, the resin was mixed uniformly and then subjected to viscosity testing.

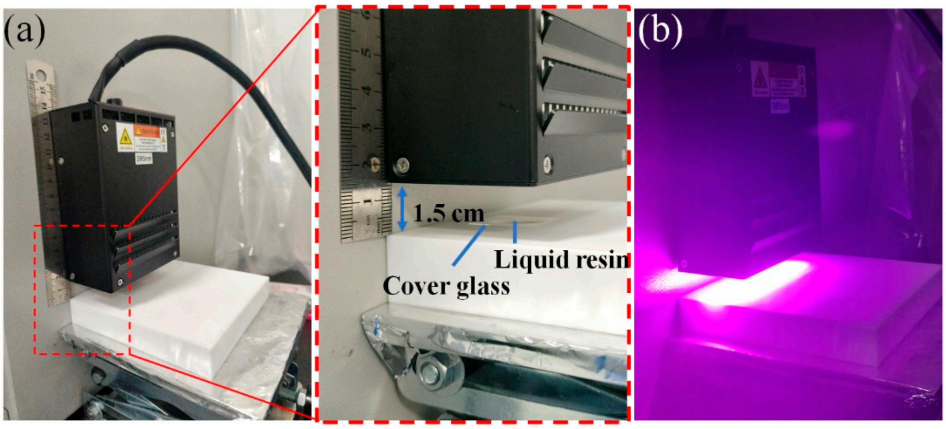

Figure S2. Photos of the UV-curing resin device: (a) The UV-curing device and sample placement details, and (b) the UV-curing device during operation.

3. Volatilization experiment

Experiment 1: A certain mass of each of the two resins was weighed and placed in an oven at 25 °C. The samples were removed at 30-minute intervals for weighing and recording over a total duration of 2 hours.

Experiment 2: A certain mass of each of the two resins were respectively weighed and placed in an oven at 60 °C. The samples were removed at 15-minute intervals for weighing and recording over a total duration of 1 hour. The elevated temperature was employed to accelerate the chemical reactions. At 60 °C, distinct phenomena were observed: the Elium sample began transitioning from colorless to pale yellow at the 45-minute mark. After 1 hour, the Elium sample underwent explosive polymerization, while the epoxy sample cured due to internal overheating.

4. Experiment of epoxy impregnated flax yarn

Combining the schematic diagram and the photos of the epoxy-impregnated yarn, it was evident that an increase in pulling speed introduces more air as the flax yarn enters the impregnation chamber. This air becomes entrapped in the vortex surrounding the yarn and subsequently infiltrates the yarn's interior. Numerous white bubbles were observed near the liquid surface along the chamber wall, indicating a significant increase in bubble formation within the epoxy resin. Some of these bubbles eventually migrated to the solution's surface.

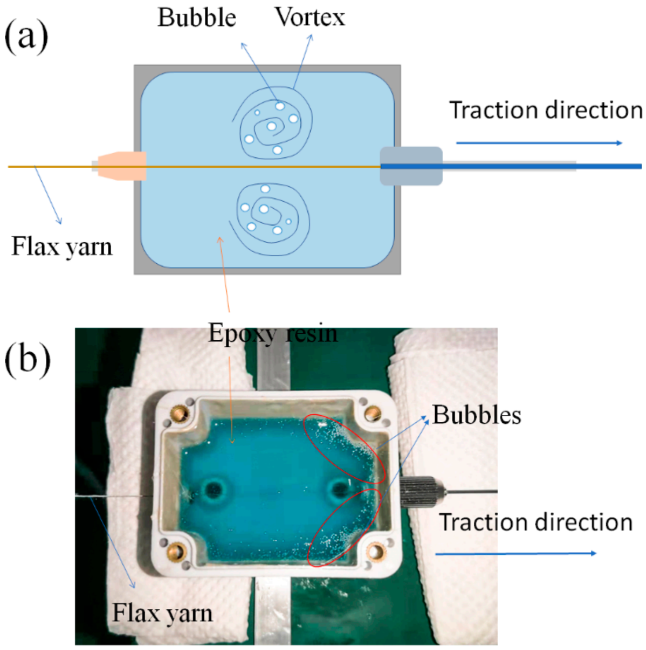

Figure S3. (a)The schematic diagram of epoxy impregnated flax yarn in the impregnated box, (b)the photo of epoxy impregnated flax yarn in the impregnated box.

5. Comparison of the deviation in size between composite and pure PLA samples

To accurately measure the length of the composite samples, we ignored the resin-rich areas at both ends of the printed samples and only measured the regions containing flax yarn (Figure S4(a)). As observed from Figure S4(b), pure PLA exhibits the best

dimensional stability, while PEIFCs demonstrate better dimensional stability compared to PEpFCs samples. This is attributed to the incompatibility between the epoxy and PLA interface, which leads to increased dimensional deviations in the printed samples. From Figure S4(c) and Table S2, it can be seen that in terms of length, PEIFCs and PEpFCs are approximately 5 % and 10 % smaller than pure PLA samples, respectively. In terms of width, PEIFCs and PEpFCs are approximately 11 % and 10 % larger than pure PLA samples, respectively. Regarding height, PEIFCs and PEpFCs are approximately 10 % and 14 % larger than pure PLA samples, respectively.

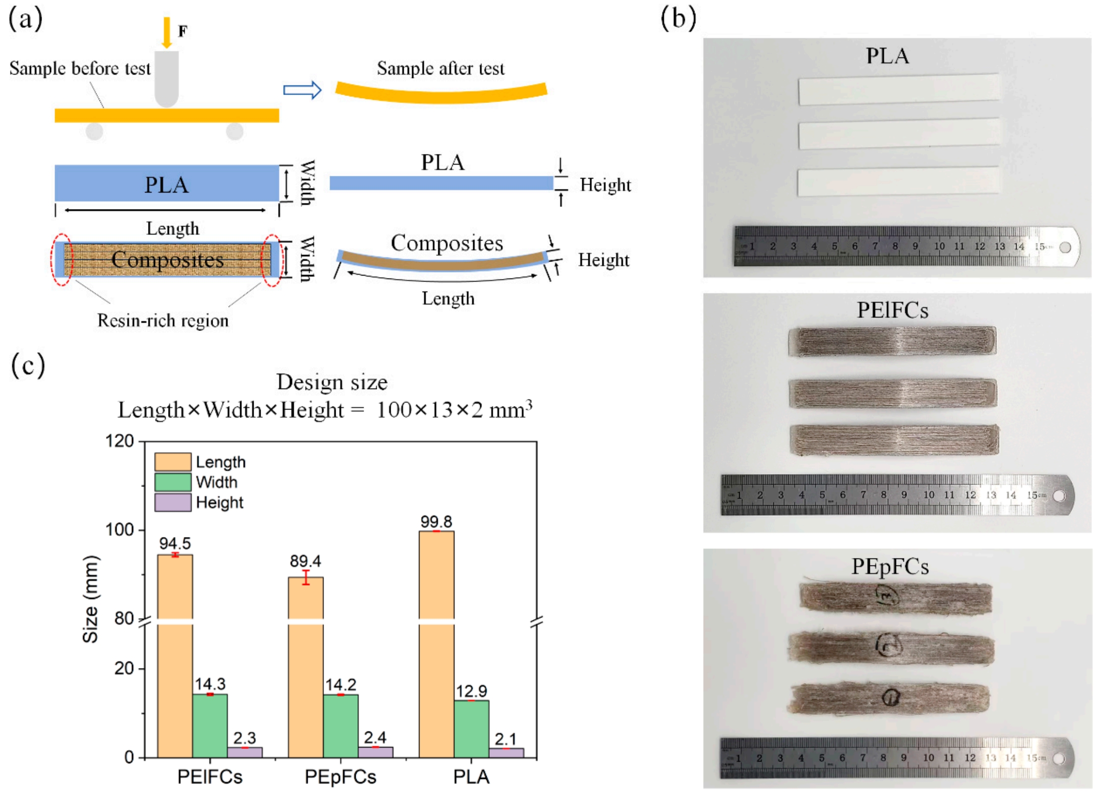

Figure S4. (a) Schematic diagram illustrating the dimensional measurement locations for PEIFCs, PEpFCs and PLA samples. (b) The photos of PEIFCs, PEpFCs and PLA samples. (c) Comparison of sizes among PEIFCs, PEpFCs and PLA samples.

Table S2. The deviation in size of PElFCs and PEpFCs compared to pure PLA and design size.

	PElFCs			PEpFCs		
	L (%)	W (%)	H (%)	L (%)	W (%)	H (%)
Pure PLA	5	11	10	10	10	14
Design size	6	10	15	11	9	20

Note：Length = L，Width = W and Height = H.
